# Central Nervous System Tuberculosis With Shower Like Pattern of Intracranial Tuberculomas in an Immunocompetent Patient

**DOI:** 10.7759/cureus.9922

**Published:** 2020-08-21

**Authors:** Saeed Arif, Shaheer Arif, Atiq-ur-Rehman Slehria, Ghulam Yousaf, Khurram Haq Nawaz

**Affiliations:** 1 Neurology, Pak-Emirates Military Hospital, Rawalpindi, PAK; 2 Radiology, Armed Forces Insititute of Radiology and Imaging, Rawalpindi, PAK; 3 Internal Medicine, Pak-Emirates Military Hospital, Rawalpindi, PAK

**Keywords:** central nervous system tuberculosis, immunocompetent patient, ring enhancement, shower like pattern, tuberculoma, tuberculous meningitis

## Abstract

Central nervous system tuberculosis (CNS TB), though comparatively uncommon as compared to other forms of extrapulmonary tuberculosis (TB), results in high morbidity and mortality. The symptoms are non-specific and of a progressive nature resulting in delayed diagnosis. We present a case of CNS TB that presented two months after the onset of symptoms. The patient’s condition reached the point of being bedbound. On investigation, the widespread involvement of the brain with shower-like pattern of ring enhancing tuberculomas and associated tuberculous meningitis was found. This was a surprising finding considering the patient was immunocompetent. Contrast-enhanced magnetic resonance imaging showed lesions in midbrain, pons, medulla, thalamus, bilateral cerebellar hemispheres and bilateral cerebral hemispheres. Upon treatment initiation the patient responded well with resolution of all lesions.

## Introduction

Tuberculosis (TB) contributes extensively to worldwide morbidity and mortality. Pakistan is fifth among countries with the highest TB burden. Central nervous system (CNS) involvement accounts for 4.6% of extrapulmonary TB cases in Pakistan. CNS TB has a treatment success rate of 74.3% in Pakistan [1] which is relatively low as compared to other forms of TB. Therefore it is prudent to detect and treat on time in order to obtain better outcomes.

CNS manifestations of TB can be tuberculous meningitis (TBM), tuberculoma, tuberculous abscess, and cerebritis. Magnetic resonance imaging (MRI) with contrast is the gold standard for radiological analysis of CNS TB. TBM is the most common manifestation [2]. CNS involvement in TB is five times greater in human immunodeficiency virus (HIV) positive individuals compared to healthy people [3]. Tuberculomas may present with focal neurological deficit, lacking systemic signs and symptoms [4]. Concurrent presence of TBM, on the other hand, may manifest with fever, headache, nausea, vomiting, neck stiffness, and seizures [2]. Extensive CNS involvement is usually suspected in immunocompromised patients; however, we came across a case of TBM with innumerable intra-cerebral tuberculomas in an immunocompetent patient.

## Case presentation

A 65-year-old male, hypertensive for five years, presented to the outpatient department with complaints of anorexia, fever, headache and weight loss for the last two months. Abnormal behavior had been present for one month. The onset of symptoms began with anorexia with the patient losing 20 kg of body weight. The patient had an intermittent fever which was followed by headache after two weeks. The headache was persistent, moderate in intensity, and involved the whole cranium with increased intensity over the occiput. The patient developed increasing amount of weakness resulting in him becoming bedbound. He also had a one-week history of confusion with difficulty in comprehension and irrelevant speech. There was no history of night sweats, cough or hemoptysis. Past medical and surgical history was insignificant. The patient was not an addict and there was no family history of TB.

Inspection revealed an emaciated male with oral thrush. Neurological examination showed confusion with disorientation in place and time. His pupils were reactive to light, stiffness of neck was present and kernig sign was positive. Fundoscopy revealed a bilateral grade two papilledema. Cranial nerve examination was unremarkable. Deep tendon reflexes were exaggerated and the tone was increased, with power reduced on medical research council scale to 3/5 in all limbs. The rest of the general physical and systemic examination including respiratory, gastrointestinal and cardiovascular examination was unremarkable.

**Figure 1:**
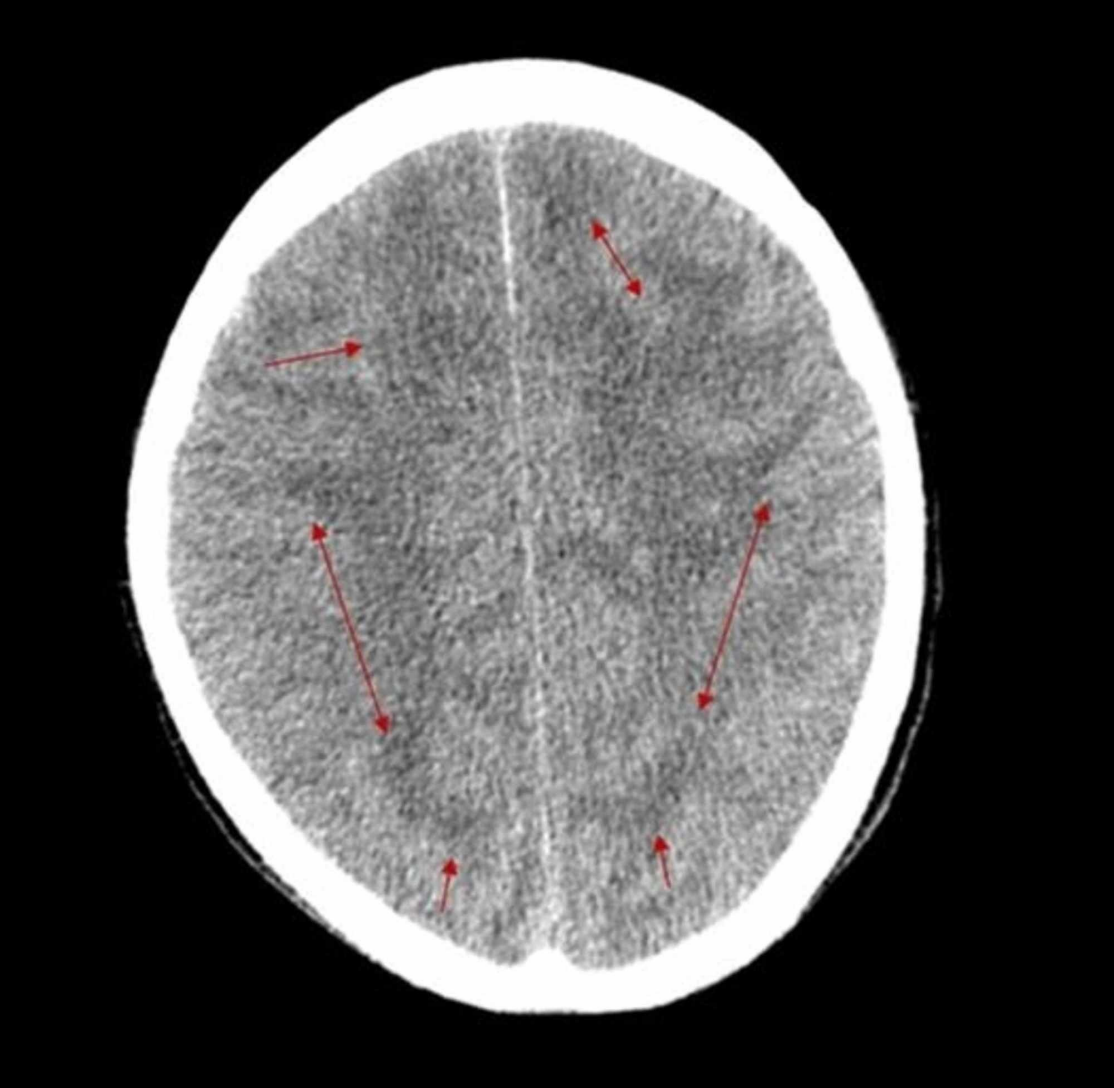
Axial image of non-contrast CT scan brain Extensive edema is visible in both cerebral hemispheres (red arrows). CT, computed tomography

**Figure 2:**
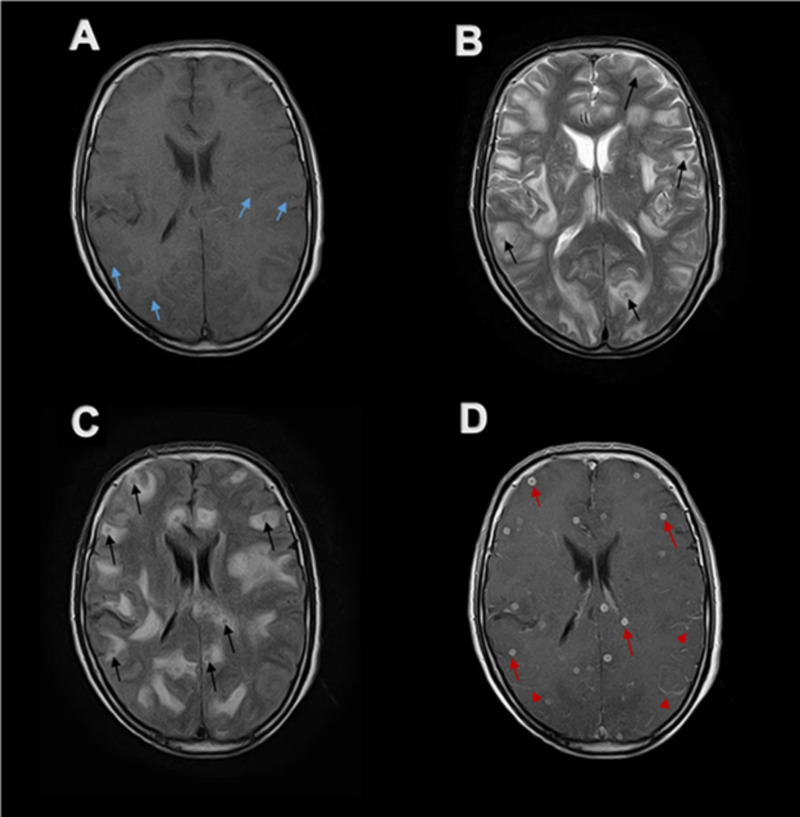
Axial MR imaging of brain at the level of basal ganglia Multiple small lesions are distributed in shower like pattern throughout the brain involving all lobes bilaterally with extensive perilesional edema. (A) Lesions appear as isointense to hypo-intense on axial T1-weighted MR image (blue arrows). (B and C) Same lesions appear hypo-intense on axial T2-weighted and FLAIR MR images (black arrows). (D) Ring enhancement is shown on post-contrast axial T1-weighted MR image (red arrows) with associated patchy gyriform leptomeningeal enhancement (red arrow heads). MR, magnetic resonance. FLAIR, fluid-attenuated inversion recovery

**Figure 3:**
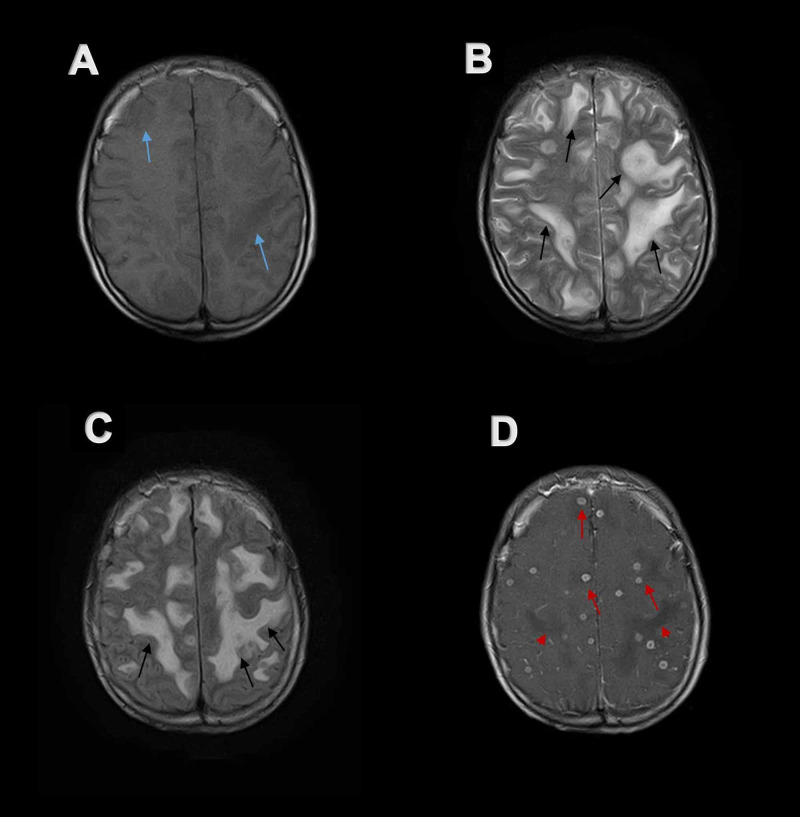
Axial MR imaging of the brain at the level of corona radiata Numerous small focal lesions are visible scattered all over the brain with extensive perilesional edema in all images. (A) Perilesional edema appears hypo-intense on axial T1-weighted MR image (blue arrows). (B, C) Perilesional edema shows hyper-intense signal (black arrows) while tuberculomas are hypo-intense on axial T2-weighted MR image and FLAIR MR image. (D) Post-contrast axial T1-weighted MR image demonstrates ring enhancing tuberculomas (red arrows) in shower like pattern with hypo-intense perilesional edema (red arrow heads). MR, magnetic resonance. FLAIR, fluid-attenuated inversion recovery

**Figure 4:**
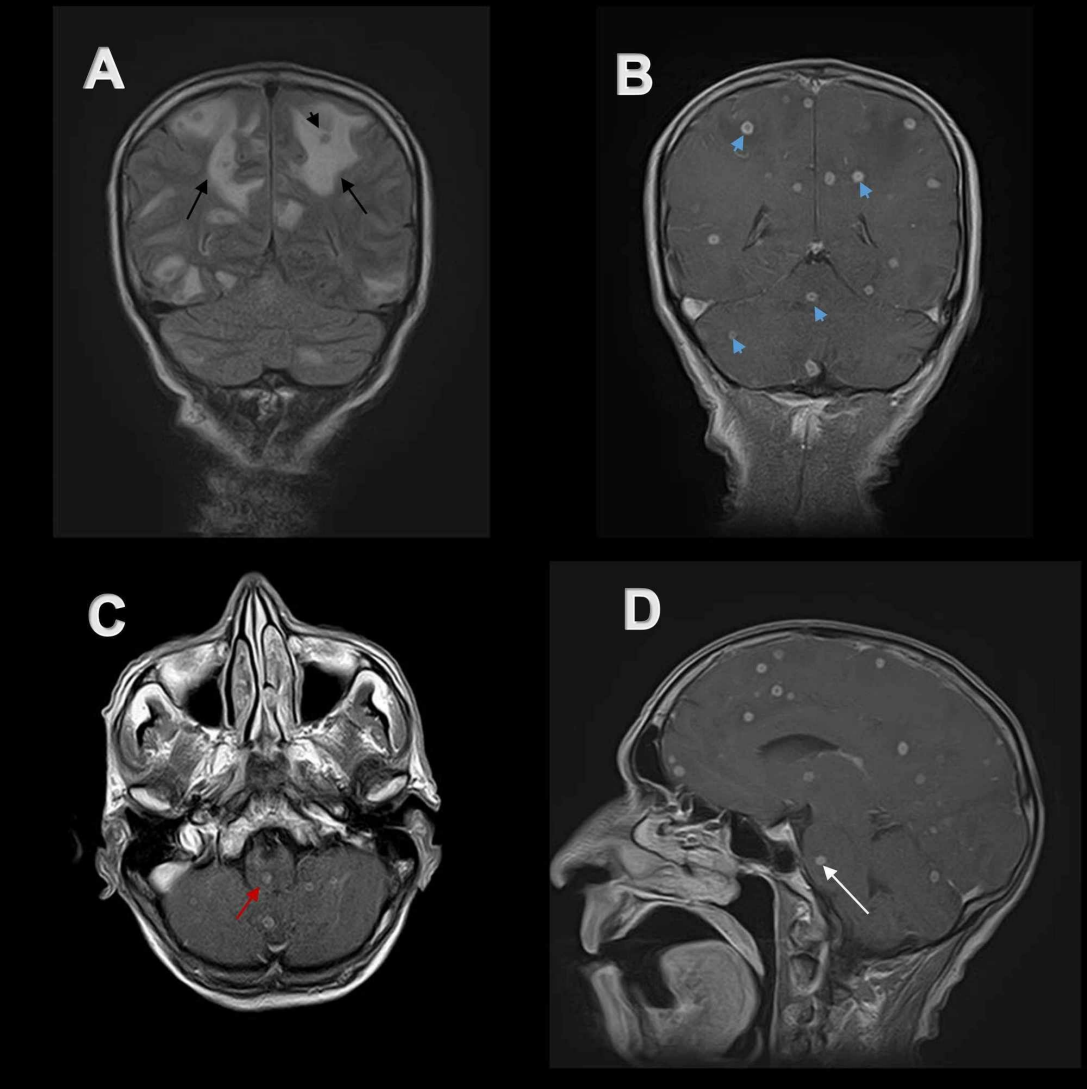
FLAIR and post-contrast T1-weighted MR images of the brain (A) Coronal FLAIR MR image of brain depicts extensive perilesional edema (black arrows) around hypo-intense lesions (black arrow head). (B) Multiple contrast enhancing lesions (blue arrow heads) are visible on post-contrast coronal T1-weighted MR image scattered throughout the brain. (C) Single lesion in medulla (red arrow) and multiple lesions in cerebellum show contrast uptake on post-contrast axial T1-weighted MR image. (D) Single ring enhancing lesion is present in pons (white arrow) on post-contrast sagittal T1-weighted MR image. MR, magnetic resonance. FLAIR, fluid-attenuated inversion recovery

**Figure 5:**
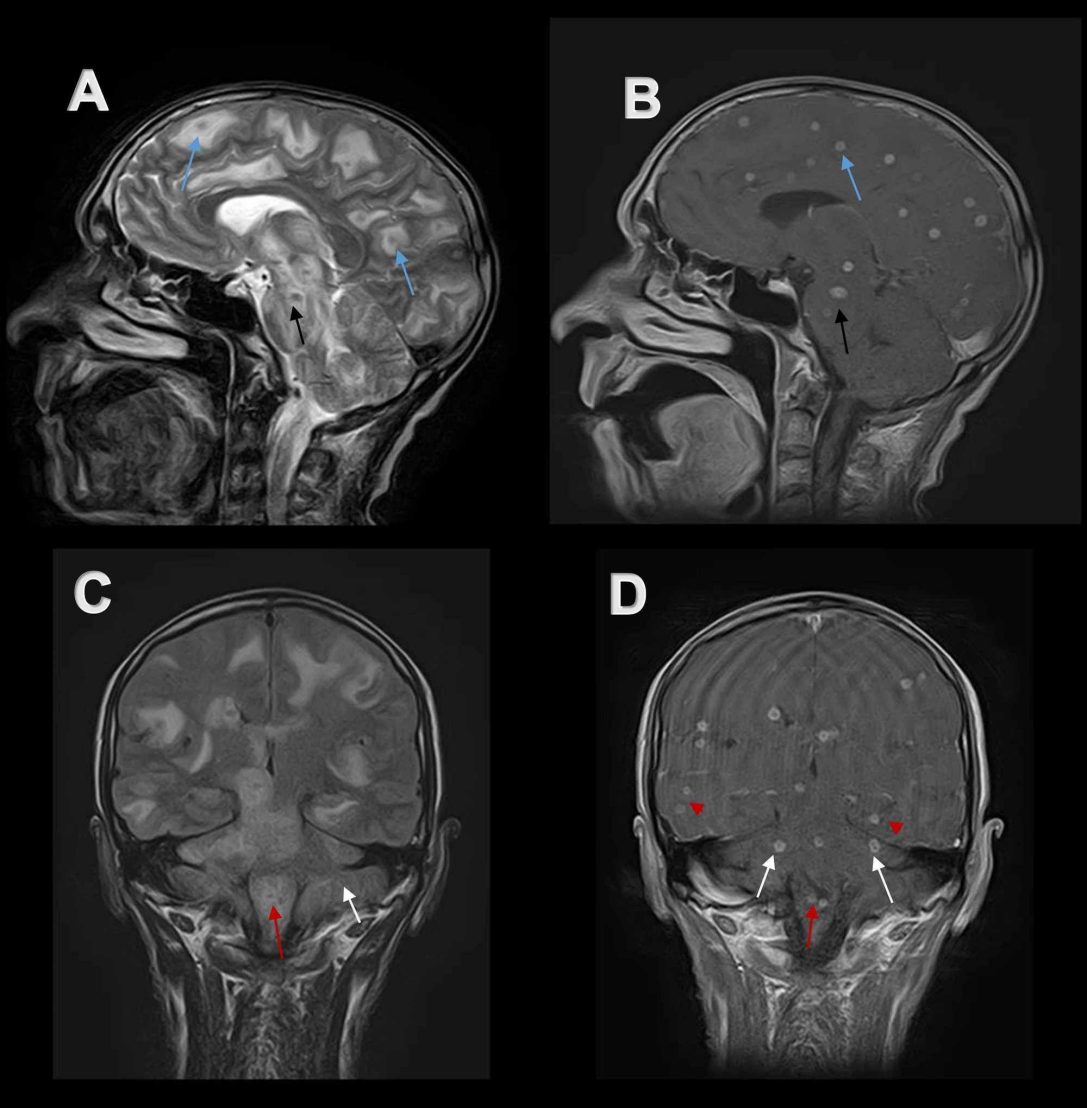
Sagittal and coronal MR imaging of the brain showing the extent of involvement (A) Sagittal T2-weighted MR image and (B) post-contrast sagittal T1-weighted MR image exhibit numerous lesions in upper brainstem (black arrows) and cerebral hemisphere (blue arrows). (C) Coronal FLAIR MR image and (D) post-contrast T1-weighted coronal MR image show widespread lesions in the lower brain stem (red arrows), bilateral temporal lobes (red arrow heads), and cerebellum (white arrows). MR, magnetic resonance. FLAIR, fluid-attenuated inversion recovery

**Figure 6:**
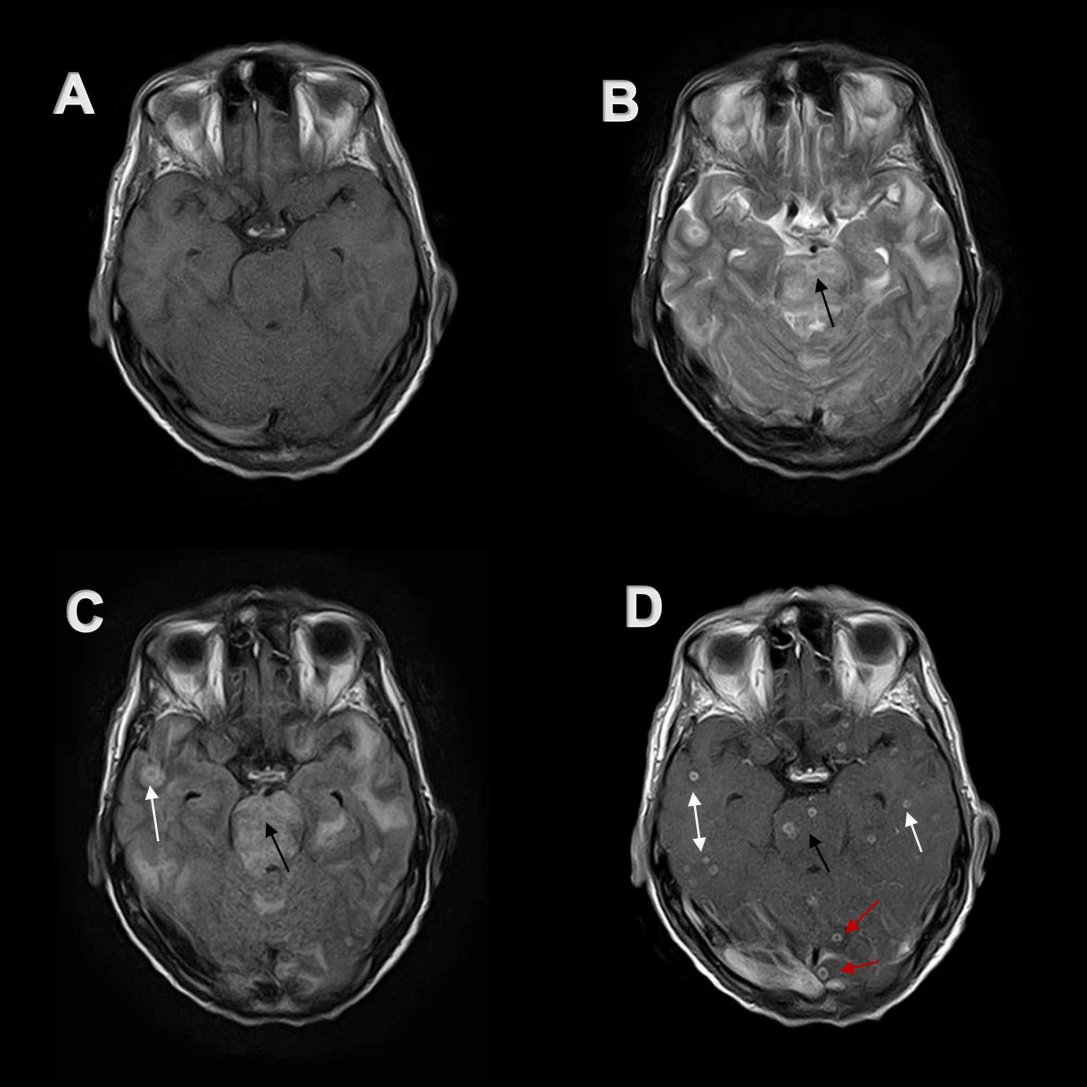
Axial MR imaging of the brain at the level of pons Numerous lesions are present in bilateral temporal lobes (white arrows) and occipital lobes (red arrows) along with two lesions in the pons (black arrows). (A) Lesions are isointense on axial T1-weighted MR image. (B and C) Same lesions appear hypo-intense on axial T2-weighted MR image and axial FLAIR MR image. (D) These lesions show avid ring contrast enhancement on post-contrast axial T1-weighted MR image. MR, magnetic resonance. FLAIR, fluid-attenuated inversion recovery

**Figure 7:**
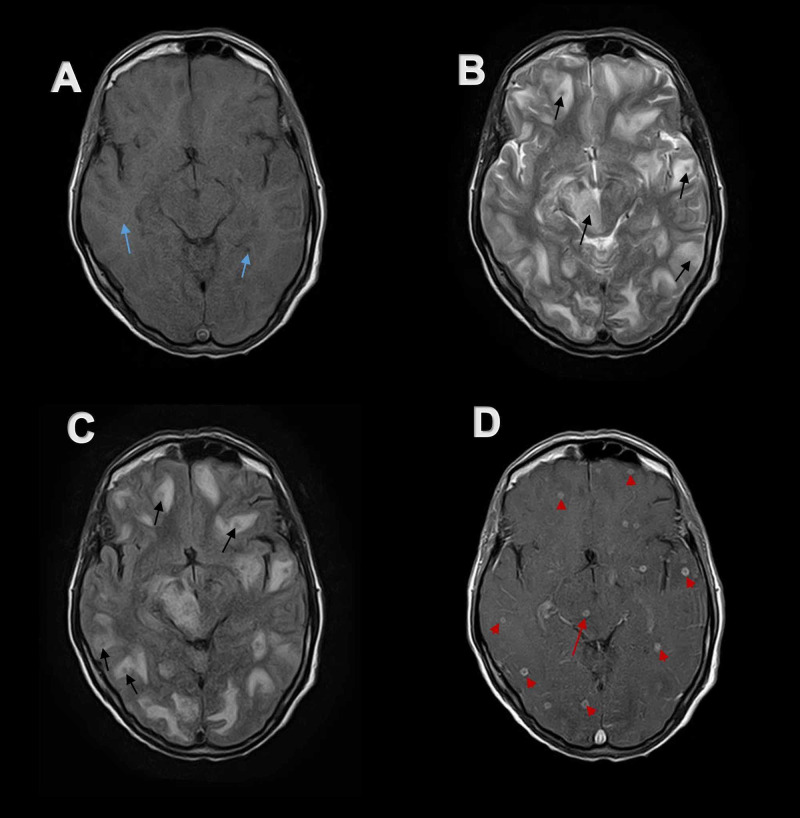
Axial MR imaging of the brain at the level of midbrain Multiple small focal lesions are dispersed throughout the brain. (A) Lesions appear isointense to hypo-intense (blue arrows) on axial T1-weighted MR image. (B and C) These lesions demonstrate hypo-intense signal (black arrows) on axial T-2 weighted MR image and axial FLAIR MR image. (D) Same lesions show peripheral contrast uptake on post-contrast axial T1-weighted MR image with central hypo-intense signal (red arrow heads) demonstrating ring enhancement. A single lesion in the right midbrain (red arrow) is also visible. MR, magnetic resonance. FLAIR, fluid-attenuated inversion recovery

Computerized tomography (CT) of brain without contrast (Figure 1) showed hypodense areas with extensive perilesional edema bilaterally in cerebral hemispheres, giving an impression of space-occupying lesions in the brain. Contrast-enhanced magnetic resonance imaging (CE MRI) of the brain showed innumerable widespread lesions in the occipital, frontal, parietal and temporal lobes, involving both cerebral hemispheres (Figures 2, 3), right thalamus (Figure 4D) and splenium of the corpus callosum. Lesions were also present in bilateral cerebellar hemispheres and vermis (Figures 4, 5). Moreover, the brainstem showed extensive involvement including medulla (Figure 4C), pons (Figures 5A, 5B, 4D, 6), and right midbrain (Figure 7).

**Figure 8:**
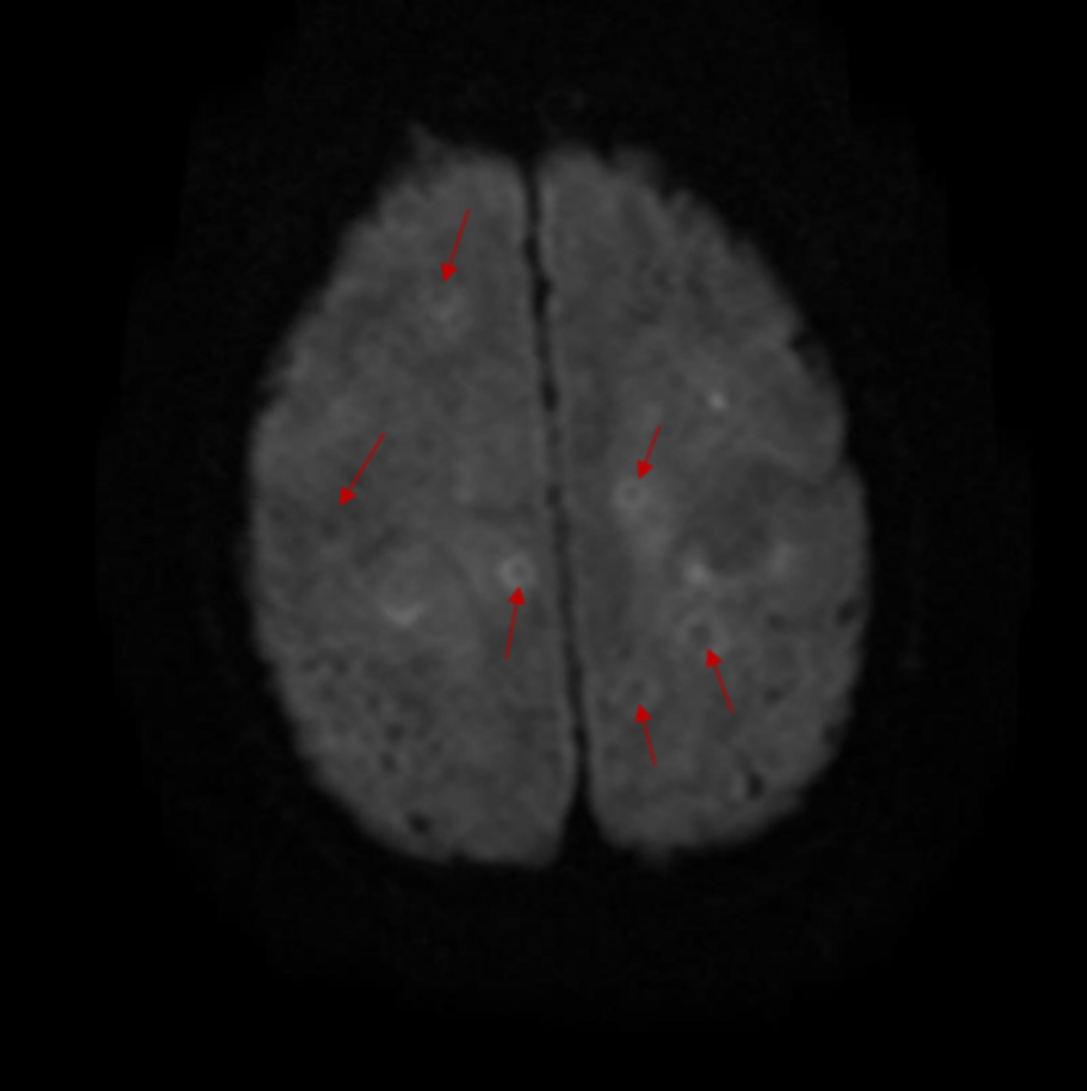
Axial DW MR imaging of the brain Multiple lesions are seen with central portion of these lesions showing low-intensity signal (red arrows). This finding is not consistent with restricted diffusion. DW, diffusion weighted. MR, magnetic resonance

Lesions were isointense to hypo-intense on T1-weighted magnetic resonance (MR) images (Figures 2A, 3A, 6A, 7A). Lesions on T2-weighted and fluid-attenuated inversion recovery (FLAIR) MR images were centrally hypo-intense and had peripheral high-intensity signal (Figures 2B, 2C, 3B, 3C, 4A, 5A, 5C, 6B, 6C, 7B, 7C). These lesions were surrounded by perilesional edema which appeared hyper-intense on T2-weighted and FLAIR MR images while showed hypo-intense signal on plain T1-weighted and post-contrast T1-weighted MR images. On post gadolinium contrast T1-weighted MR images, lesions showed extensive ring enhancement with hypo-intense signals in the center, distributed throughout the brain in shower like pattern (Figures 2D, 3D, 4B-4D, 5B, 5D, 6D, 7D). The central hypo-intense signal did not show restricted diffusion on diffusion-weighted MR images (Figure 8). CE MRI spinal cord showed only mild meningeal enhancement.

**Figure 9:**
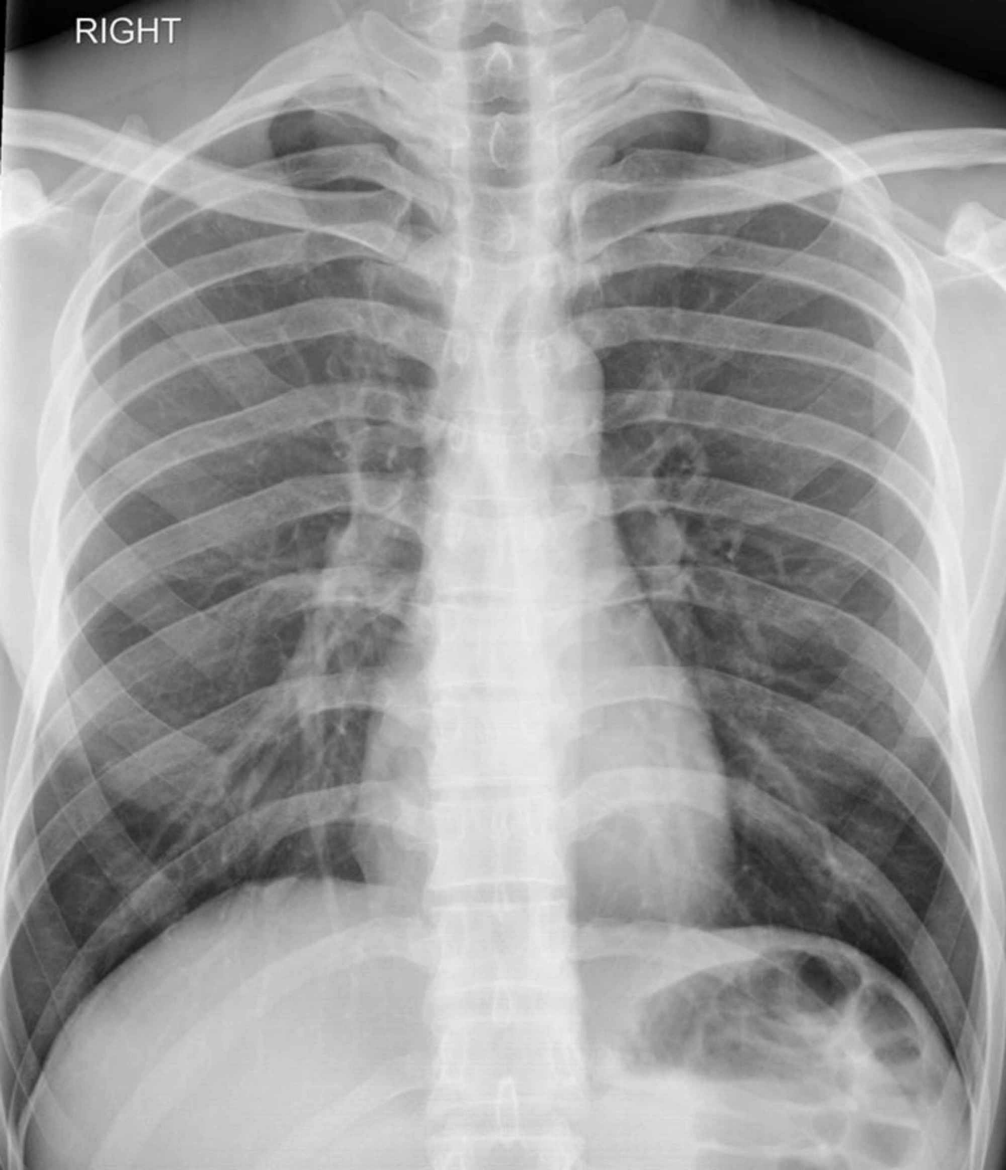
X-ray chest AP view showing normal lung fields AP, anteroposterior

**Figure 10:**
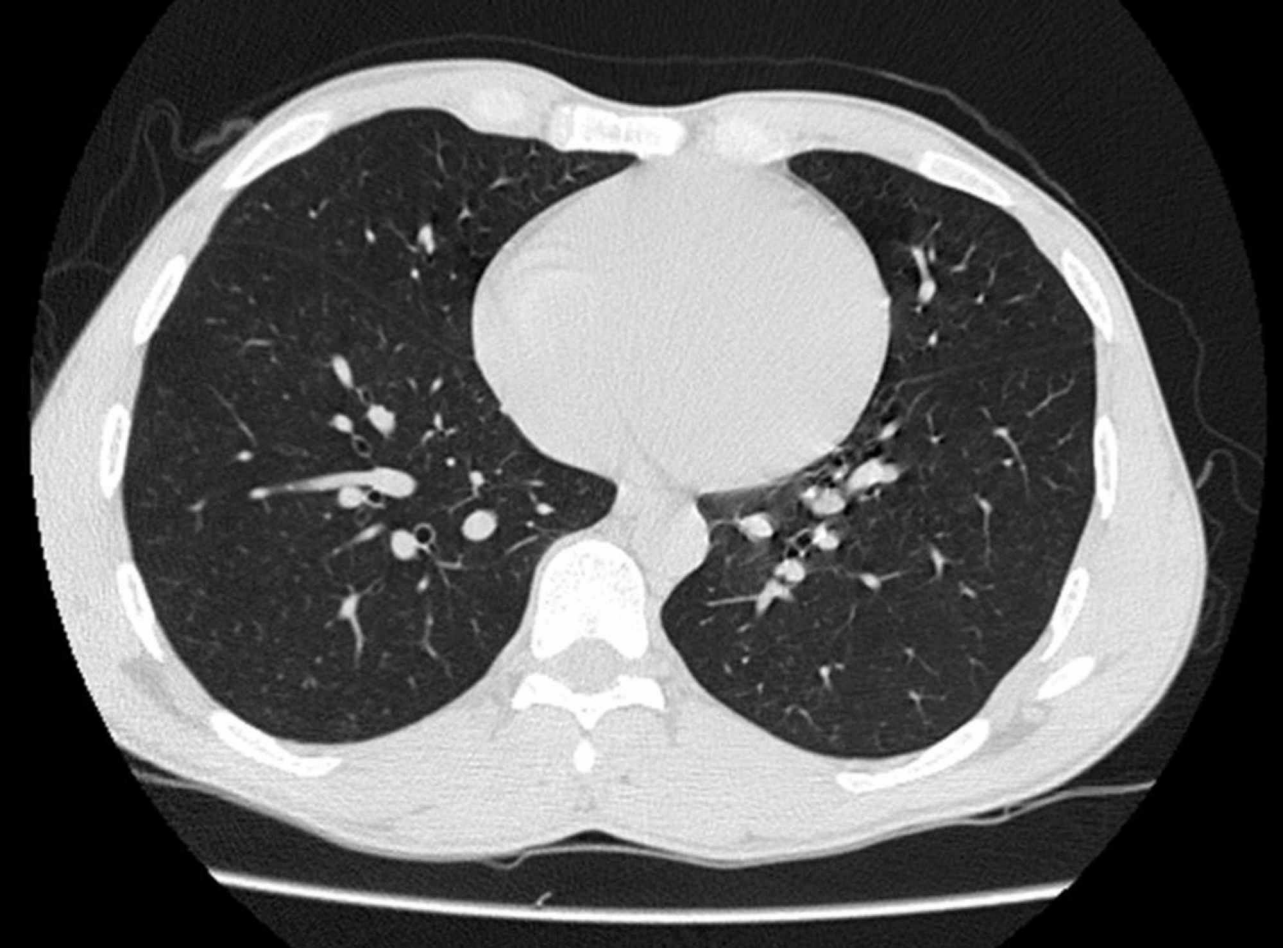
Axial image of high-resolution CT scan of chest in lung window at the level of heart reveals no abnormality in lung fields CT, computerized tomography

**Figure 11:**
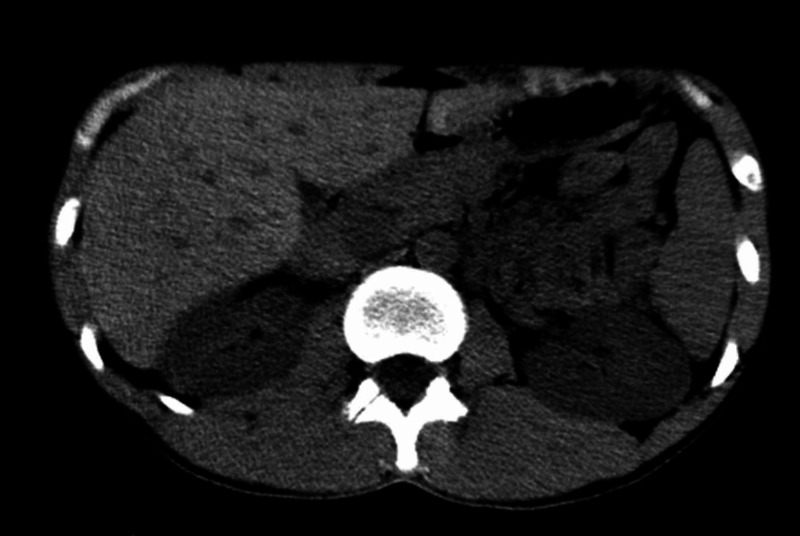
Axial image of CT scan of abdomen in abdominal window at the level of liver revealing no abnormality in visualized viscera CT, computerized tomography

Keeping in view the raised intracranial pressure, cerebrospinal fluid (CSF) analysis was performed after CE MRI of the brain. Opening pressure was 230 mmH2O, protein was 135 mg/dl, glucose was 32 mg/dl, and white blood cells (WBC) were 250/µl with predominance of lymphocytes. Acid-fast bacillus (AFB) staining was negative, while polymerase chain reaction (PCR) for Mycobacterium tuberculosis (MTB) came out positive with MTB strain being sensitive to isoniazid and rifampicin. CSF immunocytochemistry did not reveal any atypical cells. Moreover, the cryptococcal antigen was also negative in CSF. The chest X-ray was normal (Figure 9). High-resolution CT of chest (Figure 10), and contrast-enhanced CT of abdomen and pelvis (Figure 11) did not show any abnormality.

Laboratory investigations revealed an erythrocyte sedimentation rate of 54. Both HIV serology and PCR for HIV RNA were negative. Moreover, serology for toxoplasma and cysticercosis was also negative. The autoimmune screen including anti-nuclear antibodies, anti-ds-DNA antibodies, anti-thyroid antibodies and extractable nuclear antibodies were unremarkable. Based on clinical history, examination, and MRI brain findings, diagnosis of TB meningitis with CNS tuberculomas was made.

The patient was started on intravenous dexamethasone and an extended regimen of anti-tuberculosis therapy including intravenous moxifloxacin, rifampicin, isoniazid, ethambutol and pyrazinamide for TBM, along with an anti-epileptic drug. The patient's condition started improving with treatment, and moxifloxacin was stopped after two weeks. After two months, only rifampicin and isoniazid were continued for 10 months. Steroids were continued for two months with tapering doses. The patient recovered quite well with resolution of confusion and behavioral symptoms after going through one month of inpatient treatment. The patient was able to walk without support with final power 4/5 in all limbs on discharge. The patient's MRI brain repeated after completion of anti-tuberculosis therapy showed complete resolution of lesions, with the patient becoming clinically symptom-free.

## Discussion

CNS involvement is a relatively rare presentation of tuberculosis as compared to other sites of extrapulmonary TB [1]. It is the gravest systemic presentation of TB due to the increased mortality rate, with regular neurological complications and permanent deficits [5]. It usually presents with non-specific symptoms of headache, fever, neck rigidity, focal neurological deficits, alteration in consciousness, and behavioral changes [6]. It most commonly involves children and HIV-affected individuals, although there is significant occurrence in immunocompetent adults as well [7].

To confirm the diagnosis of CNS TB, there must be either evidence of TB in CSF or histopathological confirmation of caseating granuloma with or without identification of AFB [2]. However, CSF analysis is not done in all cases, and even when done, may come out negative. Stereotactic brain biopsy is not readily available in developing countries, so treatment is undertaken on probable diagnosis as well. In our patient CSF analysis came out positive for MTB PCR, hence a confirmed diagnosis was made. However, differential diagnoses were ruled out.

In cases of intra-cerebral tuberculomas, the most common location is supratentorial, while brain stem involvement accounts for only 2.5 to 8 percent of cases [2, 8]. In patients with nervous system involvement of TB, 50 percent have presence of TB outside the nervous system as well [5, 9]. This may serve as an important clue for diagnosis but, as in our case, may be absent. Usually, in the case of CNS involvement in an immunocompetent host, the involvement of the brain is not extensive [10]. In our case, there was a shower-like spread of tuberculomas throughout the brain which is unusual.

Treatment of CNS TB is recommended as a quadruple regimen for 12 months in uncomplicated cases. The drugs included are isoniazid, rifampicin, pyrazinamide and ethambutol. Ethambutol can be switched with streptomycin [11]. Other drugs like fluoroquinolones and linezolid can also be given in the initial intensive phase of disease depending upon severity of disease and resistance to the standard anti-tuberculous drugs. Steroids are included in the regimen for the first six to eight weeks [12]. Our patient went through treatment for 12 months, and after completion, repeat CE MRI showed no enhancing lesion. The patient, despite having initial extensive CNS involvement, was clinically symptom-free.

## Conclusions

CNS TB is a difficult diagnosis to make, and carries high mortality and morbidity. Clinical presentation of this disease is non-specific with a wide range of differentials of typical radiological findings. This makes fast and accurate diagnosis a tedious process. Nonetheless, timely diagnosis is essential for improved patient outcomes.

Our case report sheds light on the magnitude of involvement that can occur in TB of CNS affecting the entire brain in an immunocompetent individual, and appropriate therapy can lead to resolution of symptoms and complete recovery.
